# Long intergenic non-coding RNA -00917 regulates the proliferation, inflammation, and pyroptosis of nucleus pulposus cells via targeting miR-149-5p/NOD-like receptor protein 1 axis

**DOI:** 10.1080/21655979.2022.2043100

**Published:** 2022-02-19

**Authors:** Tengfei Li, Ye Peng, Yufei Chen, Xiaogang Huang, Xiaojie Li, Zhenyu Zhang, Junjie Du

**Affiliations:** Department of Orthopedics, Air Force Medical Center of Pla, Beijing, Haidian District, China

**Keywords:** Intervertebral disc degeneration, LINC00917, pyroptosis, inflammation

## Abstract

Intervertebral disc degeneration (IDD) has caused great trouble in people’s lives. Dysregulated long noncoding RNAs (lncRNAs) are closely linked to IDD progression. Our study aims to analyze the role of LINC00917 in the progression of IDD. Forty nucleus pulposus (NP) IDD tissues and 40 NP tissues of intervertebral discs without apparent degeneration were collected. TBHP was used to induce IDD. Cell proliferation was measured using the MTT and EdU assays. Pyroptosis was detected using flow cytometry. RT-qPCR and Western blot assays were performed to determine mRNA, miRNA, and protein expression. Dual-luciferase reporter and RNA pull-down assays were performed to verify the relationship between LINC00917 or NLRP1 and miR-149-5p. LINC00917 expression was enhanced in TBHP-treated nucleus pulposus cells (NPCs). The knockdown of LINC00917 promoted proliferation and inhibited cytotoxicity, inflammatory response, and pyroptosis of NPCs. LINC00917 functions as a sponge for miR-149-5p. Having silenced miR-149-5p, the effects of LINC00917 knockdown on NPC proliferation and inflammation-induced pyroptosis were alleviated. NLRP1 overexpression induced cellular dysfunction and pyroptosis of NPCs. LINC00917 knockdown restored NPC cellular functions and inhibited IDD progression by modulating the miR-149-5p/NLRP1 axis.

## Graphical Abstract

**Figure:**
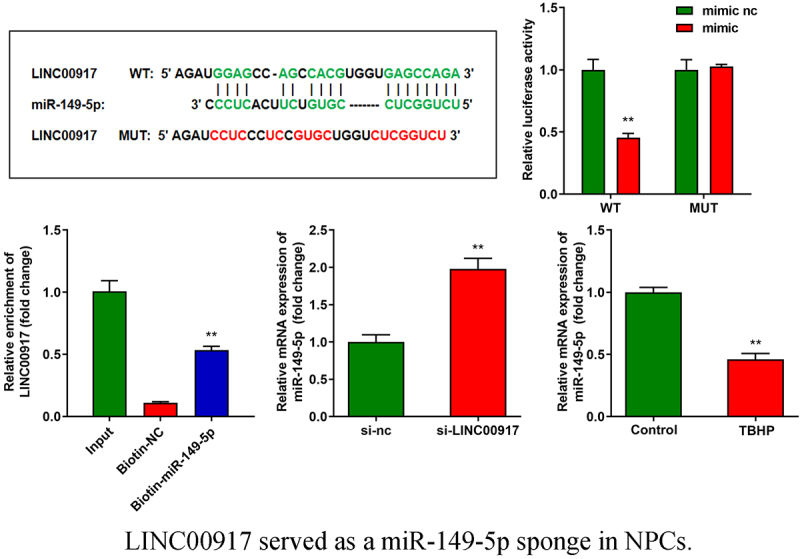

## Introduction

In general, intervertebral disc degeneration (IDD) induces the development of lower back pain and shoulder and neck pain [1]. IDD is characterized by changes in the proteoglycan state, loss of bound water molecules, and decrease in tissue osmotic pressure, which induce changes in the mechanical action of the intervertebral disc and ultimately cause pain in a specific area [2]. The typical symptom of IDD is lower back pain; approximately 75–85% of the elderly suffer from lower back pain induced by IDD [3]. This significantly affects the quality of life and work efficiency and increases the expenditure of medical care [4]. In addition, previous studies demonstrated that IDD can lead to neurological deficits and disability [5,6]. Currently, cytokine/protein injection, cell transplantation, and gene transfer are three potential strategies for the treatment of IDD [^7–9^]. However, the existing side effects neutralize the clinical outcomes. Therefore, it is important to explore an effective method for treating IDD.

IDD is usually caused by programmed cell death of nucleus pulposus cells (NPCs), because NPCs are essential for maintaining the mechanical and biochemical homeostasis of the intervertebral disc [10]. Recent studies have illustrated that when IDD occurs, NPCs would form inflammasomes and die via pyroptosis [11,12]. It has been reported that pyroptosis is more dangerous than apoptosis, because pyroptotic cells release a lot of inflammatory factors, which would lead to aggravation of the inflammatory response [13]. Hence, inhibition of pyroptosis may be an effective method for IDD treatment.

In recent years, the potential of long-chain non-coding RNAs (lncRNAs) for IDD treatment has attracted increasing attention [14,15]. LncRNAs are important gene regulators in eukaryotic cells. LncRNAs regulate gene expression by modulating gene transcription, epigenome regulation, protein coding gene translation, and genome defense; on the other hand, lncRNAs participate in biological processes, including cell growth and metabolism [16,17]. Previous studied revealed that numerous lncRNAs are dysregulated in degenerative nucleus pulposus (NP) [18,19]. Chen et al. [20] found that 135 lncRNAs were upregulated and 170 were downregulated in IDD samples, among which LINC00917 was the most significantly differentially expressed lncRNA and suspected to modulate IDD progression. However, there was no further evidence to support this conjecture.

Therefore, in this study, we predicated and confirmed that LINC00917 functions as a miR-149-5p sponge and NLRP1 is targeted by miR-149-5p via bioinformatic tools. We aimed to explore the specific action mechanism of LINC00917 in IDD. In addition, we hypothesized that LINC00917 participates in the proliferation and pyroptosis of the TBHP treated NPCs via regulating miR-149-5p/NLRP1 axis.

## Material and methods

### Bioinfomatic analysis

The target miRNA of LINC00917 was predicted by in online starBase software (http://starbase.sysu.edu.cn/). Besides, the target gene of miR-149-5p was predicted in online TargetScan software (http://www.targetscan.org/vert_71/).

### Tissues samples

The 40 NP tissues of the intervertebral disc degeneration (IDD group) patients (sex, 23 male and 17 female; mean age, 24.3 ± 5.1; Pfirrmann grade IV) and 40 NP tissues of intervertebral disc without apparent degeneration patients (sex, 18 male and 22 female; mean age, 22.5 ± 4.2; Pfirrmann grade I or II) were collected from idiopathic scoliosis surgery in Air Force Medical Center of PLA. All patients agreed to our experiments and signed the informed consent form. Our research was approved by the ethics committee of Air Force Medical Center of PLA.

### Cell isolation and transfection

Next, according to the previous study [21], we collected the NP tissues, cut them into 1 mm^3^ pieces, and placed them in a centrifuge tube. Trypsin (0.25%) was added to the tubes for digestion at 37°C in water and centrifuged for 20 min. Next, the supernatant was discarded and type-II collagenase was added. NPCs were maintained in the DMEM-F12 medium supplemented with 10% FBS and 5% CO2 at 37°C. To establish the IDD model *in vitro*, the NPCs were cultured with 100 μM TBHP for 4 h. NPCs from the control group were added to the same amount of culture medium.

Cells in the logic growth phase were transfected with siRNA targeting LINC00917 (si-LINC00917 1#, si-LINC00917 2#), miR-149-5p mimic and inhibitor, overexpression NLRP1 (oe-NLRP1), and their controls (Genepharma, China) using Lipofectamine® 3000 reagent (Invitrogen, USA). si-LINC00917 1#: 5’-CUUUCUGUCACAUUGACCACCCUG-3’; si-LINC00917 2#: 5’-UAGCACCAUUUGAAAUCAGUGUU-3’.

### RT-qPCR

TRIzol (Beyotime, Nantong, China) was used to separate RNA. Nanodrop1000 (Invitrogen, USA) was used to analyze the purity and concentration of the extracted RNA to ensure 8 ≤ OD260/OD280 ≤ 2.0. The diluted RNA was reverse-transcribed using a reverse transcription kit (Takara, Dalian, China). The reverse transcription parameters were as follows: reaction at 37°C for 15 min, 3 cycles; denatured at 85°C for 5 s. Next, the SYBR® Premix Ex Taq™ quantitative kit was used to perform RT-qPCR. The reaction conditions were as follows: pre-denaturation at 95°C for 30s, 40 cycles; denaturation at 95°C for 5 s and annealing at 60°C for 30s. GAPDH was used as the internal reference. The 2^−ΔΔCt^ method was used to quantify the relative expression according to a previous study [22].

### MTT

According to a previous study [23], NPCs in each group were seeded in 96-well plates at a density of 1 × 10^4^ cells/well and cultured for twenty-four hours. Then, the cell was treated with MTT for four hours. Next, the cells were treated with dimethyl sulfoxide. Finally, optical density was measured at 490 nm using a microplate reader (Thermo Scientific, CA, USA).

### EdU

According to a previous study [24], NPC cells (4000 cells/well) were seeded in 96-well plates. Next, 50 μM EdU solution (KeyGEN, Nanjing, China) was added. After culturing for one hour, the NPCs were washed, and 100 μL PBS containing 4% paraformaldehyde was added for 30 min. Next, NPCs were incubated with 2 mg/mL glycine and 100 μL PBS (containing 0.5% Triton X-100). Subsequently, NPCs were incubated with 1 mg/mL DAPI for 10 min. Finally, a fluorescence microscope (Thermo Scientific) was used to observe the five randomly selected fields, and the number of EdU-positive cells was quantified.

### Determination of cell pyroptosis

According to a previous study [25], NPCs (1 × 10^6^ cells/ml) were seeded into 24-well plates. The cells were then washed and resuspended. After that, the cells were incubated with 5 ml μl Hoechst33342 and 5 μl PI in the dark for 15 min. Finally, the pyroptotic cells were analyzed by flow cytometry (BD Biosciences, USA).

### Western blot

According to a previous study [26], radioimmunoprecipitation assay lysis buffer was used to separate the proteins from the NPCs. The protein concentration was analyzed using a BCA Kit (Beyotime). Then, 60 μg of protein was selected to conduct the twelve alkyl sulfate polyacrylamide gel electrophoresis. The proteins were then transferred to a PVDF membrane. The membranes were blocked and incubated with primary antibodies overnight at 4°C (anti-NLRP3, 1:600; anti-GADMD-N, 1:1000; Caspase1, 1:800; GAPDH, 1:2000; Abcam, USA). The membranes were then probed with an HRP-conjugated secondary antibody. Next, the membranes were exposed and imaged using ECL detection reagents (Thermo Scientific). The ImageJ software (version. 1.8.0, National Institutes of Health) was used to quantify proteins.

### Dual-Luciferase reporter assay

According to a previous study [27], the wild-type (WT) and mutant (MUT) 3′ UTR regions of LINC00917 and NLRP1 were cloned into the pmiR-RB-Report™ vector (Promega). The miR-149-5p mimic and the WT and MUT 3′ UTR regions of LINC00917 or NLRP1 were co-transfected into the NPCs. The dual-luciferase reporter assay system (Promega) was used to measure luciferase activity.

### RNA pull-down assay

According to a previous study [28], the NPCs were treated with biotinylated miR-149-5p or NC probes. After transfection for 48 h, the NPCs were treated with cell lysate (Ambion, Austin, TX, USA) for 10 min. The cleavage was incubated with beads precoated with M-280 streptavidin (Sigma) at 4°C for 3 h. Then, the bound RNA was purified using Trizol (Boyetime), and LINC00917 or NLRP1 levels were measured by RT-qPCR.

### Animal experiments

The 18 male mice were provided by the Air Force Medical Center of PLA. Before the beginning of the experiment, all mice were adaptively raised in the animal room for 3 days, conventionally raised alone, and free to food and drink. Then the mice were randomly divided into 3 groups with 6 mice in each group: Sham group, Model+LV-sh-NC group and Model+LV-sh-LINC00917 group. The specific puncture methods for IDD establishment were as follows: The mice were injected 10% chloral hydrate intraperitoneally to anesthesia. After that, the C2-C6 supraspinous ligament and interspinous ligament of the mice were removed in turn, the skin was sutured after hemostasis. After the operation, intramuscular injection of penicillin 8 × 104 U was administered every day for 3 consecutive days. The mice in the Sham group were only sutured after incision of the skin to stop the bleeding. Seven days after modeling, the 20 μL LV-sh-NC or LV-sh-LINC00917 (107 particles/μL) were intravenously injected via the tail vein into designated group to knockout the LINC00917 expression. After four weeks, the mice were euthanized and intervertebral disc tissues were collected. The animal studies were approved by the ethics committee of Air Force Medical Center of PLA.

### Determiantion of the Basso Beattie Bresnahan (BBB) score, paw withdrawal latency and paw withdrawal threshold

Before the mice in each group were killed, the mice were placed in a transparent observation box and counted with a stopwatch. From the contact between the hind foot and the tray to the emergence of tiptoe, retraction, licking and struggle, the latency of hot stimulation foot contraction reflex was recorded, and paw withdrawal latency and paw withdrawal threshold were measured. Additionally, the BBB score were also recorded according to a previous study [29].

### HE staining

According to a previous study [30], all mice were killed under excessive anesthesia with 3% Pentobarbital Sodium. Then the back skin of the mcie was cut and the intervertebral disc tissue was stripped. The C4-C5 intervertebral disc specimens of mice were taken and fixed in 40 g/L paraformaldehyde solution. Then the tissues were cut into 5 μM section and stained with hematoxylin eosin. Finally, the section was observed under optical microscope.

### Determination of LDH, IL-6, IL-1β, and TNF-α levels

According to a previous study [31], the LDH, IL-6, IL-1β, and TNF-α levels of the NPCs and tissues were detected according to the instructions of the matched test kit provided by the Nanjing Jiangcheng Bioengineering Institute (Nanjing, China).

### Statistical analysis

The data were analyzed using SPSS 21.0. The Student’s t-test was used to analyze the differences between the two groups. One-way analysis of variance was performed for comparisons between multiple groups. The results are expressed as mean ± SD. Where p smaller than 0.05 means that the difference is statistically significant.

## Results

In this study, LINC00917 expression was up-regulated in IDD tissues and TBHP treated NPCs. However, knockdown of LINC00917 promoted the proliferation, and inhibited the inflammatory response and pyroptosis of NPCs via regulating miR-149-5p/NLRP1 axis.

### LINC00917 up-regulated in IDD

**Figure 1.**
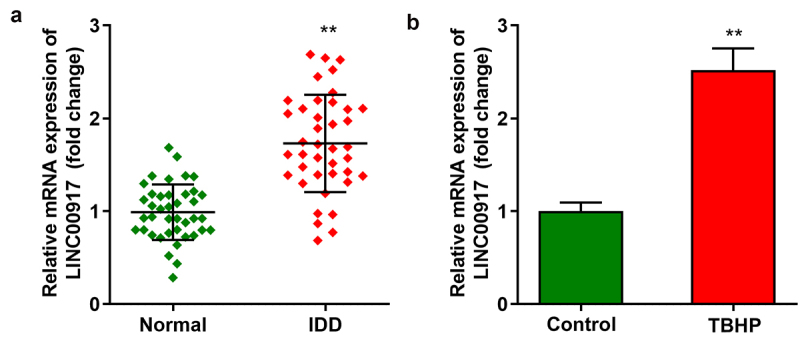
**LINC00917 was up-regulated in IDD. A** The LINC00917 expression levels in IDD tissues. **B** The LINC00917 expression in NPCs. **P < 0.01.

First, we demonstrated that LINC00917 levels were dramatically elevated in the IDD tissues (Figure 1(a)). In addition, LINC00917 was dramatically overexpressed in TBHP-treated NPCs (Figure 1(b)).

### LINC00917 knockdown promoted proliferation and relieved pyroptosis and inflammation in TBHP-treated NPCs

**Figure 2.**
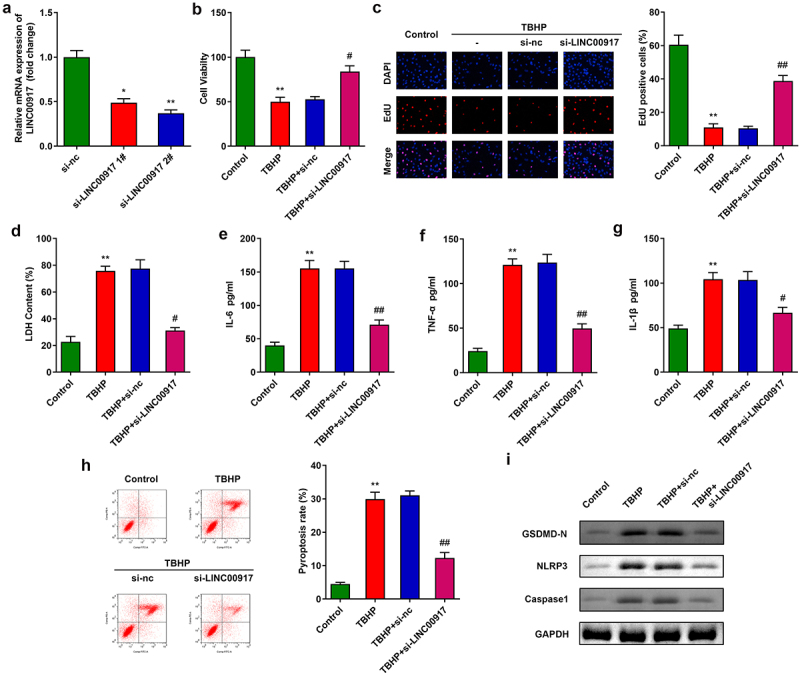
**Knockdown of LINC00917 promoted proliferation, and relieved the pyroptosis and inflammation of the TBHP treated NPCs. A** Validation of transfection efficiency. **B-C** MTT and EdU assays were performed to measured the proliferation. **D-G** The LDH, IL-6, TNF-α, and IL-1β levels were determined with corresponding kits. **H** The pyroptosis rate was detected with flow cytometry. **I** The protein expressions of NLRP3, GSDMD-N and Caspase1. *P < 0.05, **P < 0.01 VS Control group; #P < 0.05, ##P < 0.01 VS TBHP+si-nc group.

Next, after transfection with si-LINC00917 1# and si-LINC00917 2#, LINC00917 expression was dramatically downregulated in NPCs (Figure 2(a)). According to transfection efficiency, si-LINC00917 2# was applied for further experiments. Knockdown of LINC00917 significantly promoted NPC proliferation (Figure 2(b–c)). In addition, knockdown of LINC00917 significantly decreased cytotoxicity and the release of pro-inflammatory cytokines such as IL-6, TNF-α, and IL-1β (Figure 2(d–g)). Furthermore, LINC00917 knockdown alleviated the cell death of NPCs induced by TBHP and the pyroptosis-related proteins NLRP3, GSDMD-N, and Caspase1 (Figure 2(h,i)).

### LINC00917 sponge to miR-149-5p sponge in NPCs

**Figure 3.**
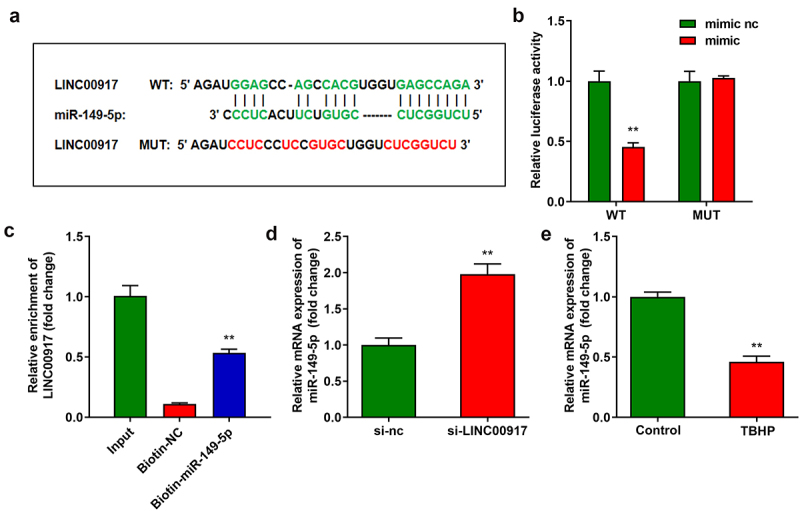
**LINC00917 served as a miR-149-5p sponge in NPCs. A** Binding sites between miR-149-5p and LINC00917 predicted by starBase. **B-C** The relationship between LINC00917 and miR-149-5p verified by dual-luciferase reporter and RNA pull-down assays. **D** The miR-149-5p expression detected after si-LINC00917 transfection. **E** The miR-149-5p expression levels in NPCs. **P < 0.01.

Using online starBase software, miR-149-5p was predicted to target LINC00917 (Figure 3(a)). The binding sites were verified by luciferase and RNA pull-down assays. (Figure 3(b-c)). In addition, the knockdown of LINC00917 significantly increased miR-149-5p expression (Figure 3(d)). Moreover, miR-149-5p expression was significantly downregulated in IDD cells (Figure 3(e)).

### NLRP1 targeted to miR-149-5p

**Figure 4.**
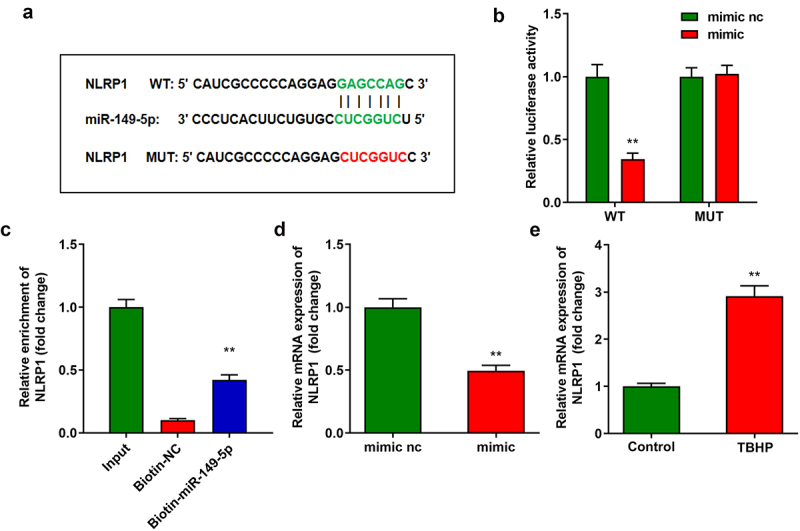
**NLRP1 is targeted by miR-149-5p. A** Binding sites between miR-149-5p and NLRP1 predicted by TargetScan. **B-C** Dual-luciferase reporter and RNA pull-down assays conducted to verify the relationship between NLRP1 and miR-149-5p. **D** The NLRP1 expression detected by RT-qPCR. **E** The NLRP1 expression in NPCs. **P < 0.01. **P < 0.01.

Figure 4(a) displays the binding sites between NLRP1 and miR-149-5p (Figure 4(a)), which were confirmed by luciferase and RNA pull-down assays (Figure 4(b-c)). In addition, the overexpression of miR-149-5p significantly decreased NLRP1 expression (Figure 4(d)). NLRP1 expression was significantly increased in the IDD tissues (Figure 4(e)).

### LINC00917 function regulation of NPCs via miR-149-5p/NLRP1 axis

**Figure 5.**
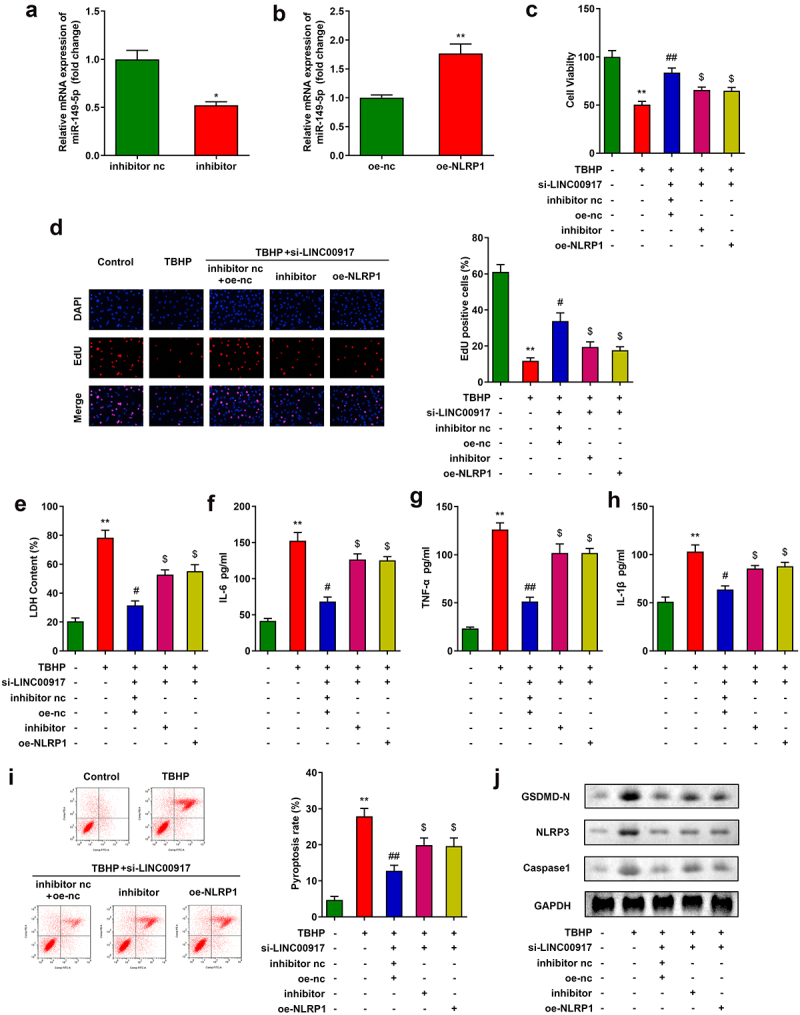
**LINC00917 regulated the functions of the NPCs via miR-149-5p/NLRP1 axis. A-B** Validation of transfection efficiency. **C-D** MTT and EdU assays were performed to measured the proliferation. **E-H** The LDH, IL-6, TNF-α, and IL-1β levels determined with corresponding kits. **I** The pyroptosis rate detected with flow cytometry. **J** The protein expressions of NLRP3, GSDMD-N and Caspase1 measured by Western blot. *P < 0.05, **P < 0.01 VS Control group; #P < 0.05, ##P < 0.01 VS TBHP group; $P < 0.05 VS TBHP+si-LINC00917+ inhibitor nc+oe-nc group.

To analyze the specific mechanism of LINC00917 in IDD, we designed and transfected miR-149-5p inhibitor and oe-NLRP1 into NPCs. After transfection, miR-149-5p levels were depleted and NLRP1 levels were enhanced (Figure 5(a–b)). MTT and EdU assays showed that downregulation of miR-149-5p or overexpression of NLRP1 alleviated the functions of si-LINC00917 in NPC growth (Figure 5(c–d)). In addition, downregulation of miR-149-5p or overexpression of NLRP1 increased cytotoxicity and promoted the inflammatory response of NPCs (Figure 5(e–h)). Furthermore, the miR-149-5p inhibitor or overexpression of NLRP1 reversed the effect of si-LINC00917 on the pyroptosis rates of NPCs and the protein expression of pyroptosis-related genes, such as NLRP3, GSDMD-N, and Caspase1 (Figure 5(j)).

### Knockdown of LINC00917 relieved the IDD progression in vivo

**Figure 6.**
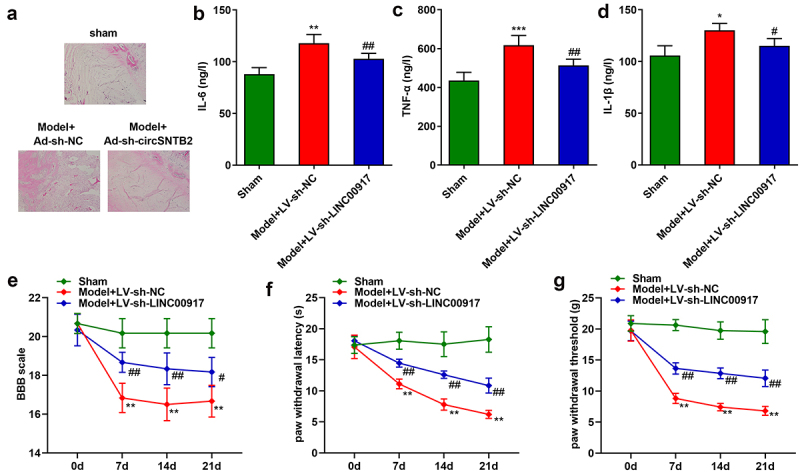
**Knockdown of LINC00917 relieved the IDD progression in vivo. A** HE staining for IDD mice. **B-D** The IL-6, TNF-α, and IL-1β levels were determined with corresponding kits. **E-G** The determination of mouse behavior. *P < 0.05, **P < 0.01, *P < 0.001 VS Sham group; #P < 0.05, ##P < 0.01 VS Model+LV-sh-NC group.

Last, we explore the role of LINC00917 in the IDD mice. The results of HE staining showed that in the sham group, the nucleus pulposus tissue of the mice was normal. In the Model group, the nucleus pulposus tissue is deformed, the fibrous rings are arranged disorderly, and the matrix rich in mucopolysaccharide and cell clusters penetrate each other. These phenomena were alleviated after LINC00917 knockout (Figure 6(a)). Besides, the IL-6, TNF-α, and IL-1β were increased in the Model group, while LINC00917 knockout decreased them (Figure 6(b-d)). Additionally, in the Model group, the BBB score, paw withdrawal latency and paw withdrawal threshold were significantly decreased, while LINC00917 knockout increased them (Figure 6(e-g)).

## Discussion

In the current study, LINC00917 expression was upregulated in IDD tissues and TBHP-treated NPCs. However, the knockdown of LINC00917 promoted proliferation and inhibited the inflammatory response and pyroptosis of NPCs by regulating the miR-149-5p/NLRP1 axis.

Pyroptosis is inflammatory programmed cell death mediated by two cysteine aspartate proteases (caspases): classical Caspase1 and non-classical caspase4/5/11. There are five inflammasomes that can stimulate pyroptosis: NLRP1, NLRP3, NLRC4, IPAF, and AIM2 of the NLR family. The classical pyroptosis pathway is mainly induced by the activation of caspase1, which is activated by the NLR family inflammasome [32]. Activated Caspase1 promoted the production of Contents (LDH) and inflammatory factors (IL-1β and IL-18), which are released into the extracellular environment [33]. Simultaneously, Gasdermin-D (GSDMD) is lysed to form an active fragment GSDMD-N with a pore-forming effect to induce cell pyroptosis [34]. Previous research expounded that pyroptosis is related to the IDD development [11,12]. Here, we confirmed that the protein expression of Caspase1, NLRP3, and GSDMD-N and the LDH, TNF-α, IL-1β, and IL-6 levels were upregulated in TBHP-treated NPCs, implying that pyroptosis occurs in IDD, suggesting that degeneration of the intervertebral disc is accompanied by inflammatory death or pyroptosis of NPCs. Therefore, the Achilles heel of IDD may be to inhibit NPC pyroptosis.

Recently, the role of lncRNAs in the development of IDD has attracted extensive attention. Accumulating evidence has demonstrated that significant changes occur in the expression of many lncRNAs in IDD. Wan et al. [35] confirmed that 116 lncRNAs were significantly altered in IDD. Chen et al. [20] revealed that LINC00917 participates in IDD progression. Similarly, LINC00917 expression was enhanced in IDD tissues and in TBHP-treated NPCs. In addition, the knockdown of LINC00917 promoted proliferation and inhibited pyroptosis (a form of inflammation-related cell death) of NPCs. These findings imply that LINC00917 acts as a promoter of IDD.

lncRNAs can function as sponges” for miRNAs to participate in the regulation of the pathophysiological processes of IDD; that is, lncRNAs play a competitive role in endogenous RNA by targeting miRNAs. Zhang et al. [15] found that lncRNA-PART1 facilitates IDD development by modulating miR-190a-3p. Moreover, Tang et al. [36] demonstrated that lncRNA-TUG1 induces apoptosis of IDD NPCs by regulating the miR-26a/HMGB1 axis. Sun et al. [37] showed that lncRNA H19 accelerates cell death in IDD NPCs by targeting the miR-139-3p/CXCR4/NF-κB axis, thereby promoting the development of IDD. However, to the best of our knowledge, the specific mechanism of action of LINC00917 in IDD has not yet been reported. In the present study, LINC00917 was found to sponge miR-149-5p. Aberrant expression of miR-149-5p is linked to the initiation and progression of various diseases, such as atherosclerosis [38], esophageal cancer [39], and preeclampsia [40]. miR-149-5p has been confirmed as a prognostic biomarker for gliomas [41]. Overexpression of miR-149-5p inhibits the decrease in neurons and restores brain function both in vivo and in vitro [42]. In the current study, we found that downregulation of miR-149-5p reversed the effects of si-LINC00917 on NPC growth and pyroptosis. Previous studies have mainly focused on the potential of miR-149-5p in cancer [43], cardiovascular diseases [44], and bone disorders [45]. Only 2.4% of studies revealed that miR-149-5p functions in the central nervous system. Therefore, investigating the possible mechanisms of miR-149-5p in IDD is of vital importance.

miRNAs participate in IDD progression by binding to the 3’ UTR of related genes. For instance, miR-623 suppresses oxidative stress-induced apoptosis of NPCs by binding to thioredoxin-interacting protein [46]. Mesenchymal stem-cell-derived extracellular vesicle-induced upregulation suppresses IDD by inactivating LRG1 signaling [47]. In this study, miR-149-5p was found to directly target NLRP1. Qin et al. [48] revealed that NLRP1 was the first discovered pattern recognition receptor to form an inflammasome [49]. Activation of the NLRP1 inflammasome can promote the maturation and release of IL-1β, IL-6, and other inflammatory factors [50]. In this study, NLRP1 increased TNF-α, IL-1β, and IL-6 levels and promoted pyroptosis of NPCs. These findings indicated that LINC00917 regulated the growth and inflammation of TBHP-treated NPCs via the miR-149-5p/NLRP1 axis.

## Conclusion

To sum up, LINC00917 inhibits the proliferation, induced the inflammation and pyroptosis of NPCs via targeting miR-149-5p/NLRP1 axis. This study provided potential therapeutic targets for IDD.

## Data Availability

The datasets used and analyzed during the current study are available from the corresponding author on reasonable request.

## Highlights

1. LINC00917 served as a miR-149-5p sponge in NPCs.
2. LINC00917 was up-regulated in IDD.
3. LINC00917 regulated the functions of the NPCs via miR-149-5p/NLRP1 axis.

## References

[1] Vergroesen PP, Kingma I, Emanuel KS, et al. Mechanics and biology in intervertebral disc degeneration: a vicious circle. Osteoarthritis Cartilage. 2015;23(7):1057. 25827971 10.1016/j.joca.2015.03.028

[2] Johnson ZI, Shapiro IM, Risbud MV. Extracellular osmolarity regulates matrix homeostasis in the intervertebral disc and articular cartilage: evolving role of TonEBP. MATRIX BIOL. 2014;40:10. 25172826 10.1016/j.matbio.2014.08.014 PMC4390124

[3] Schol J, Sakai D, Johnson ZI. Cell therapy for intervertebral disc herniation and degenerative disc disease: clinical trials. INT ORTHOP. 2019;43(4):1011. 30498909 10.1007/s00264-018-4223-1

[4] Navone SE, Marfia G, Giannoni A, et al. Inflammatory mediators and signalling pathways controlling intervertebral disc degeneration. Histology and Histopathology. 2017;32(6):523. 27848245 10.14670/HH-11-846

[5] Lawson LY, Harfe BD. Developmental mechanisms of intervertebral disc and vertebral column formation. Wiley Interdiscip Rev Dev Biol. 2017;6. 10.1002/wdev.283 28719048

[6] Murray CJL, Lopez AD. Measuring the Global Burden of Disease. N Engl J Med. 2013;369(5):448. 23902484 10.1056/NEJMra1201534

[7] Feng G, Zha Z, Huang Y, et al. Bioresponsive Two-Stage Delivery of Therapeutic miRNA via Polyplex Micelle-Loaded Injectable Hydrogels for Inhibition of Intervertebral Disc Fibrosis. ADV HEALTHC MATER. 2018;7(21):e1800623. 30296017 10.1002/adhm.201800623

[8] Lin H, Ma X, Wang BC, et al. Edaravone ameliorates compression-induced damage in rat nucleus pulposus cells. LIFE SCI. 2017;189:76. 28942283 10.1016/j.lfs.2017.09.024

[9] Jia Z, Yang P, Wu Y, et al. Comparison of biological characteristics of nucleus pulposus mesenchymal stem cells derived from non-degenerative and degenerative human nucleus pulposus. EXP THER MED. 2017;13(6):3574. 28588682 10.3892/etm.2017.4398 PMC5450795

[10] Zhao C, Wang L, Jiang L, et al. The cell biology of intervertebral disc aging and degeneration. AGEING RES REV. 2007;6(3):247. 17870673 10.1016/j.arr.2007.08.001

[11] Zhang J, Zhang J, Zhang Y, et al. Mesenchymal stem cells‐derived exosomes ameliorate intervertebral disc degeneration through inhibiting pyroptosis. J CELL MOL MED. 2020;24(20):11742. 32860495 10.1111/jcmm.15784 PMC7579702

[12] Zhao K, An R, Xiang Q, et al. Acid‐sensing ion channels regulate nucleus pulposus cell inflammation and pyroptosis via the NLRP3 inflammasome in intervertebral disc degeneration. CELL PROLIFERAT. 2021;54(1):e12941. 10.1111/cpr.12941 PMC7791185 33111436

[13] Man SM, Karki R, Kanneganti TD. Molecular mechanisms and functions of pyroptosis, inflammatory caspases and inflammasomes in infectious diseases. IMMUNOL REV. 2017;277(1):61. 28462526 10.1111/imr.12534 PMC5416822

[14] Tan H, Zhao L, Song R, et al. The long noncoding RNA SNHG1 promotes nucleus pulposus cell proliferation through regulating miR-326 and CCND1. American Journal of Physiology: Cell Physiology. 2018;315(1):C21 29466672 10.1152/ajpcell.00220.2017

[15] Zhang Z, Huo Y, Zhou Z, et al. Role of lncRNA PART1 in intervertebral disc degeneration and associated underlying mechanism. EXP THER MED. 2021;21(2):131. 33376513 10.3892/etm.2020.9563 PMC7751492

[16] Liu N, Wang Z, Zhao M, et al. Role of non-coding RNA in the pathogenesis of depression. GENE. 2020;735:144276. 31816363 10.1016/j.gene.2019.144276

[17] Yan M, Pan XF, Liu Y, et al. Long noncoding RNA PVT1 promotes metastasis via miR-484 sponging in osteosarcoma cells. Eur Rev Med Pharmacol Sci. 2020;24(5):2229. 32196583 10.26355/eurrev_202003_20488

[18] Cui S, Liu Z, Tang B, et al. LncRNA MAGI2-AS3 is down-regulated in intervertebral disc degeneration and participates in the regulation of FasL expression in nucleus pulposus cells. BMC MUSCULOSKEL DIS. 2020;21(1):149. 10.1186/s12891-020-3086-y PMC7059357 32143617

[19] Chen J, Jia YS, Liu GZ, et al. Role of LncRNA TUG1 in intervertebral disc degeneration and nucleus pulposus cells via regulating Wnt/beta-catenin signaling pathway. Biochem Biophys Res Commun. 2017;491(3):668. 28756222 10.1016/j.bbrc.2017.07.146

[20] Chen Y, Ni H, Zhao Y, et al. Potential Role of lncRNAs in Contributing to Pathogenesis of Intervertebral Disc Degeneration Based on Microarray Data. Med Sci Monit. 2015;21:3449. 26556537 10.12659/MSM.894638 PMC4646231

[21] Novais EJ, Tran VA, Johnston SN, et al. Long-term treatment with senolytic drugs Dasatinib and Quercetin ameliorates age-dependent intervertebral disc degeneration in mice. NAT COMMUN. 2021;12(1):5213. 34480023 10.1038/s41467-021-25453-2 PMC8417260

[22] Yang P, Liang K, Wang W, et al. LncRNA SOX2-OTinhibitionprotects against myocardialischemia/reperfusion-inducedinjury via themicroRNA-186-5p (miR-186-5p)/Yin Yang 1 (YY1)pathway. BIOENGINEERED. 2022;13(1):280. 34967264 10.1080/21655979.2021.2000229 PMC8805857

[23] Ge Z, Liu H, Ji T, et al. Long non-coding RNA 00960 promoted the aggressiveness of lung adenocarcinoma via the miR-124a/SphK1 axis. BIOENGINEERED. 2022;13(1):1276. 34738865 10.1080/21655979.2021.1996507 PMC8805815

[24] Xiao R, Wang H, Yang B. MicroRNA-98-5p modulates cervical cancer progression via controlling PI3K/AKT pathway. BIOENGINEERED. 2021;12(2):10596. 34895048 10.1080/21655979.2021.2000722 PMC8810110

[25] Zhang L, Qiu J, Shi J, et al. MicroRNA-140-5p represses chondrocyte pyroptosis and relieves cartilage injury in osteoarthritis by inhibiting cathepsin B/Nod-like receptor protein 3. BIOENGINEERED. 2021;12(2):9949. 34565303 10.1080/21655979.2021.1985342 PMC8810115

[26] Guan S, Jin T, Han S, et al. Dihydroartemisinin alleviates morphine-induced neuroinflammation in BV-2 cells. BIOENGINEERED. 2021;12(2):9401. 34854364 10.1080/21655979.2021.1982311 PMC8810002

[27] Clement T, Salone V, Rederstorff M. Dual luciferase gene reporter assays to study miRNA function. Methods Mol Biol. 2015;1296:187. 25791601 10.1007/978-1-4939-2547-6_17

[28] Cheng C, Zhang H, Dai Z, et al. Circular RNA circVRK1 suppresses the proliferation, migration and invasion of osteosarcoma cells by regulating zinc finger protein ZNF652 expression via microRNA miR-337-3p. BIOENGINEERED. 2021;12(1):5411. 34424826 10.1080/21655979.2021.1965695 PMC8806728

[29] Liu X, Sun X, Shen K, et al. Aldehyde dehydrogenase 2 overexpression inhibits neuronal apoptosis after spinal cord ischemia/reperfusion injury. NEURAL REGEN RES. 2017;12(7):1166. 28852401 10.4103/1673-5374.211198 PMC5558498

[30] Lee ES, Kwon MH, Kim HM, et al. Curcumin analog CUR5-8 ameliorates nonalcoholic fatty liver disease in mice with high-fat diet-induced obesity. METABOLISM. 2020;103:154015. 31758951 10.1016/j.metabol.2019.154015

[31] Chen L, Liu P, Feng X, et al. Salidroside suppressing LPS-induced myocardial injury by inhibiting ROS-mediated PI3K/Akt/mTOR pathway in vitro and in vivo. J CELL MOL MED. 2017;21(12):3178. 28905500 10.1111/jcmm.12871 PMC5706507

[32] Zhao L, Xing R, Wang P, et al. NLRP1 and NLRP3 inflammasomes mediate LPS/ATP‑induced pyroptosis in knee osteoarthritis. MOL MED REP. 2018;17(4):5463. 29393464 10.3892/mmr.2018.8520

[33] Linton MF, Babaev VR, Huang J, et al. Macrophage Apoptosis and Efferocytosis in the Pathogenesis of Atherosclerosis. Circ J. 2016;80(11):2259. 27725526 10.1253/circj.CJ-16-0924 PMC5459487

[34] Shi J, Gao W, Shao F. Pyroptosis: gasdermin-Mediated Programmed Necrotic Cell Death. Trends in biochemical sciences (Amsterdam. Regular ed.). 2017;42(4):245 10.1016/j.tibs.2016.10.004 27932073

[35] Wan ZY, Song F, Sun Z, et al. Aberrantly expressed long noncoding RNAs in human intervertebral disc degeneration: a microarray related study. ARTHRITIS RES THER. 2014;16(5):465. 25280944 10.1186/s13075-014-0465-5 PMC4201740

[36] Tang N, Dong Y, Xiao T, et al. LncRNA TUG1 promotes the intervertebral disc degeneration and nucleus pulposus cell apoptosis though modulating miR-26a/HMGB1 axis and regulating NF-κB activation. AM J TRANSL RES. 2020;12(9):5449. 33042430 PMC7540166

[37] Sun Z, Tang X, Wang H, et al. LncRNA H19 Aggravates Intervertebral Disc Degeneration by Promoting the Autophagy and Apoptosis of Nucleus Pulposus Cells Through the miR-139/CXCR4/NF-κB Axis. STEM CELLS DEV. 2021;30(14):736. 34015968 10.1089/scd.2021.0009

[38] Ye ZM, Yang S, Xia YP, et al. LncRNA MIAT sponges miR-149-5p to inhibit efferocytosis in advanced atherosclerosis through CD47 upregulation. CELL DEATH DIS. 2019;10(2):138. 30755588 10.1038/s41419-019-1409-4 PMC6372637

[39] Li F, Zhou X, Chen M, et al. Regulatory effect of LncRNA DRAIC/miR-149-5p/NFIB molecular network on autophagy of esophageal cancer cells and its biological behavior. EXP MOL PATHOL. 2020;116:104491. 32659236 10.1016/j.yexmp.2020.104491

[40] Liu R, Wang X, Yan Q. The regulatory network of lncRNA DLX6-AS1/miR-149–5p/ERP44 is possibly related to the progression of preeclampsia. 2020;Vol. 93: Placenta:Eastbourne.34. 10.1016/j.placenta.2020.02.001 32250737

[41] Xu B, Luo X, Ning X, et al. miR-149 rs2292832 C allele enhances the cytotoxic effect of temozolomide against glioma cells. Neuroreport. 2020;31(6):498. 32243354 10.1097/WNR.0000000000001440

[42] Ghasemloo E, Oryan S, Bigdeli MR, et al. The neuroprotective effect of MicroRNA-149-5p and coenzymeQ10 by reducing levels of inflammatory cytokines and metalloproteinases following focal brain ischemia in rats. BRAIN RES BULL. 2021;169:205. 33508402 10.1016/j.brainresbull.2021.01.013

[43] Zhang X, Wang S, Wang H, et al. Circular RNA circNRIP1 acts as a microRNA-149-5p sponge to promote gastric cancer progression via the AKT1/mTOR pathway. MOL CANCER. 2019;18(1):20. 30717751 10.1186/s12943-018-0935-5 PMC6360801

[44] Nong Y, Guo Y, Gumpert A, et al. Single dose of synthetic microRNA-199a or microRNA-149 mimic does not improve cardiac function in a murine model of myocardial infarction. MOL CELL BIOCHEM. 2021;476(11):4093–4106. 34287784 10.1007/s11010-021-04227-w PMC8478849

[45] Chiu Y, Bamodu OA, Fong I, et al. The JAK inhibitor Tofacitinib inhibits structural damage in osteoarthritis by modulating JAK1/TNF-alpha/IL-6 signaling through Mir-149-5p. Vol. 151. New York: N.Y: Bone; 2021. p. 116024. 10.1016/j.bone.2021.116024 34052462

[46] Bao X, Wang Z, Jia Q, et al. HIF-1α-Mediated miR-623 Regulates Apoptosis and Inflammatory Responses of Nucleus Pulposus Induced by Oxidative Stress via Targeting TXNIP. In: OXID MED CELL LONGEV. Lai K; 2021. p. 2021. 10.1155/2021/6389568 PMC8355979 34394829

[47] Cui S, Zhang L. microRNA-129-5p shuttled by mesenchymal stem cell-derived extracellular vesicles alleviates intervertebral disc degeneration via blockade of LRG1-mediated p38 MAPK activation. J TISSUE ENG. 2021;12:1758511983. 10.1177/20417314211021679 PMC8330460 34377430

[48] Qin C, Lv Y, Zhao H, et al. MicroRNA-149 Suppresses Inflammation in Nucleus Pulposus Cells of Intervertebral Discs by Regulating MyD88. Med Sci Monit. 2019;25:4892. 31263091 10.12659/MSM.915858 PMC6618342

[49] Martinon F, Burns K, Tschopp J. The Inflammasome: a Molecular Platform Triggering Activation of Inflammatory Caspases and Processing of proIL-β. MOL CELL. 2002;10(2):417. 12191486 10.1016/s1097-2765(02)00599-3

[50] Song A, Gao B, Fan J, et al. NLRP1 inflammasome contributes to chronic stress-induced depressive-like behaviors in mice. J NEUROINFLAMM. 2020;17(1):178 10.1186/s12974-020-01848-8 PMC7281929 32513185

